# A systematic review of randomised controlled trials with adaptive and traditional group sequential designs – applications in cardiovascular clinical trials

**DOI:** 10.1186/s12874-023-02024-1

**Published:** 2023-09-07

**Authors:** Jufen Zhang, Christy Saju

**Affiliations:** 1 School of Medicine, Faculty of Health, Education, Medicine and Social Care, Anglia Ruskin University, Bishop Hall Lane, Chelmsford, CM1 1SQ U.K.; 2 School of Cardiovascular & Metabolic Health, University of Glasgow, Glasgow, U.K.

**Keywords:** Systematic review, Randomised controlled trials, Trial designs, Cardiology, Interim analysis

## Abstract

**Background:**

Trial design plays a key role in clinical trials. Traditional group sequential design has been used in cardiovascular clinical trials over decades as the trials can potentially be stopped early, therefore, it can reduce pre-planned sample size and trial resources. In contrast, trials with adoptive designs provide greater flexibility and are more efficient due to the ability to modify trial design according to the interim analysis results. In this systematic review, we aim to explore characteristics of adaptive and traditional group sequential trials in practice and to gain an understanding how these trial designs are currently being reported in cardiology.

**Methods:**

PubMed, Embase and Cochrane Central Register of Controlled Trials database were searched from January 1980 to June 2022. Randomised controlled phase 2/3 trials with either adaptive or traditional group sequential design in patients with cardiovascular disease were included. Descriptive statistics were used to present the collected data.

**Results:**

Of 456 articles found in the initial search, 56 were identified including 43 (76.8%) trials with traditional group sequential design and 13 (23.2%) with adaptive. Most trials were large, multicentre, led by the USA (50%) and Europe (28.6%), and were funded by companies (78.6%). For trials with group sequential design, frequency of interim analyses was determined mainly by the number of events (47%). 67% of the trials stopped early, in which 14 (32.6%) were due to efficacy, and 5 (11.6%) for futility. The commonly used stopping rule to terminate trials was O’Brien- Fleming-type alpha spending function (10 (23.3%)). For trials with adaptive designs, 54% of the trials stopped early, in which 4 (30.8%) were due to futility, and 2 (15.4%) for efficacy. Sample size re-estimation was commonly used (8 (61.5%)). In 69% of the trials, simulation including Bayesian approach was used to define the statistical stopping rules. The adaptive designs have been increasingly used (from 0 to 1999 to 38.6% after 2015 amongst adaptive trials). 25% of the trials reported “adaptive” in abstract or title of the studies.

**Conclusions:**

The application of adaptive trials is increasingly popular in cardiovascular clinical trials. The reporting of adaptive design needs improving.

**Supplementary Information:**

The online version contains supplementary material available at 10.1186/s12874-023-02024-1.

## Introduction

### Background

Cardiovascular disease (CVD) is a leading cause of death globally [1]. The age-adjusted CVD death rate is 217.1 per 100,000 in the US [2] and 266 per 100,000 in the UK in 2018 (Statista). This has an impact on people’s quality of life [3] and represent a major and growing socioeconomic burden. It costs the NHS £7 billion a year [4]. Despite the current advancements in the treatments for CVD, it remains the second leading cause of death in the world.

New and more effective medicines are in need to meet the demand to improve patient’s quality of life and reduce CV events. Randomized controlled clinical trials, particular phase II/III trials play an important role for developing new drugs. Trials in cardiology are normally large and take a long time to reach their primary endpoints, such as CV deaths and hospitalizations. Because of this, traditional group sequential design [5] is commonly used in the clinical trials. The design includes at least one pre-planned interim analysis with a statistical stopping rule that is defined by group sequential boundary [6–8], which allows a trial to stop early for futility, efficacy or safety reasons when sufficient evidence is reached [9]. The decision of stopping a trial early is commonly recommended by Data and Safety Monitoring Board (DSMB) after review of the results from the interim analysis based on accumulating data. Therefore, a trial with this design can potentially save money, resources and time [10, 11]. This strategy has been shown to be effective in developing and testing the efficacy and safety of medications [12], evaluating medical devices [13, 14] and accessing novel biomarkers of CV diseases [15].

However, traditional group sequential design lacks flexibility that is required to adapt to real life situations. Recently developed adaptive trials allow investigators to modify trials based on the results of interim analysis [16, 17], such as making changes to the sample size (sample size re-estimation) and combining separate phase IIb and phase III into one trial (adaptive seamless phase IIb/III design). Adaptive trials have existed for over 25 years [18]. It has been recognised as one approach that can be used to improve the success of phase III clinical trials by reducing uncertainty [19]. The design can potentially save trial resources to meet the needs of limited finding and trial participants [20], and increase the efficiency of randomized clinical trials [21].

Trials with adaptive designs have contributed significantly to the development of medical treatment, particularly in oncology [17, 22]. Recently, there is growing interest in exploring this strategy in cardiology [23, 24]. The potential use of adoptive design has been noted in clinical trials in heart failure with preserved ejection fraction (HFpEF) and acute HF syndromes for precision medicine [25]. The design has been applied in the clinical trial of HF with reduced ejection fraction for adapting sample size selection based on a Bayesian adaptive approach [26] and was also utilised in medical device trials [27] and HF biomarker trial [28]. Studies have shown that sample size re-estimation and adaptive enrichment are feasible and methodologically sound for phase III trials in cardiology [22]. Guidance on adaptive design by the US FDA has been published in 2019 (https://www.fda.gov/media/78495/download). Despite its increasing awareness, trials using adaptive design in cardiovascular clinical trials are still very limited [29–31]. The reporting of adaptive and group sequential trials were not adequate [32]. The aim of this systematic review is to explore characteristics of adaptive and traditional group sequential trials including trial design features and trial demographic characteristics in practice and to gain an understanding how adaptive and traditional group sequential designs are currently being reported, and what types of adaptive designs are commonly applied in cardiovascular randomized clinical trials.

## Methods

### Traditional group sequential and adaptive designs

Trials with traditional (or classical) group sequential design are not allowed to modify the pre-specified study criteria, such as sample size, frequency of interim analyses, length of study and trial stopping rule [33]. Because of this, in this review this design is not considered to be an adaptive design. The possibility of inflated type I error during the multiple testing due to interim analyses is commonly controlled using a stopping rule that is used to define the stopping boundaries (critical values), such as Pocock correction [6], O’Bren-Fleming correction [7], Haybittle and Peto correction [34] or the Alpha spending approach defined by Lan and DeMets [35] including Pocock-type alpha spending function and O’Brien- Fleming-type alpha spending function. The trial can be stopped early if a stopping boundary is reached.

An adaptive design clinical trial is defined by the Food and Drug Administration (FDA) as “a study that includes a prospectively planned opportunity for modification of one or more specified aspects of the study design and hypotheses based on analysis of data (usually interim data) from subjects in the study” (https://www.fda.gov/downloads/drugs/guidances/ucm201790.pdf). The modification can be made, but are not limited to, the sample size (sample size re-estimation), population (population enrichment), treatments (multi-arm multi-stage), allocation ratio (adaptive randomisation) and biomarker (biomarker adaptive) [29, 36]. Because of complex of adaptive design, statistical simulations are often involved in the trial design process to decide the statistical stopping rules. Bayesian adaptive design [37] is a popular method in adaptive trials as the method utilize Markov Chain Monte Carlo (MCMC) methods for implementing the simulation. The posterior probability in Bayesian approach is used for creating the stopping rule and allows prior knowledge of the study to be considered.

### Search strategy

The study is designed according to the Preferred Reporting Items for Systematic Reviews and Meta-analyses (PRISMA) Statement (https://prisma-statement.org/PRISMAStatement). PubMed/Medline, Embase, and Cochrane Central Register of Controlled Trials database were searched from a period of January 1980 to June 2022. ClinicalTrials.gov (https://classic.clinicaltrials.gov) was also searched. There were no language restrictions. The search terms with the four keyword groups (trial phase, RCT, trial design and cardiovascular disease) were shown in Table 1. Details search strategies are as follows.

**Table 1:** Search strategy of key words used in the study

Search strategy	
Group	keywords
Clinical trials - phase II-III	Phase IIb-IIIb, 2b-3b, 2b/3b, IIb/IIIb, 2b-3, 2b/3, IIb/III, 2–3, II-III.
Randomised controlled trials	randomiz* or randomis* or trial*
Adaptive designs/traditional group sequential design	adapt*, design* or rule
	seamless
	group sequential
	sample size re-estimation, re-estimations, sample size adjustment or sample size modification
	MAMS or multi-arm multi-stage or multi* arm or multi* stage or drop the loser or pick the winner or play the winner or two-stage adaptive
	treatment switching or treatment adaptive
	adaptive dose*.
	treatment switching or treatment adaptive
	adaptive hypothesis
	biomarker adaptive or biomarker adaptive design or biomarker or enrichment
	multiple adaptive
	interim monitoring or interim monitor or Bayesian adaptive or stopping rule* or stopping boundar*, interim
	adaptive clinical trial* or adaptive design or flexible design or adaptive method
Cardiovascular Diseases	Cardiovascular Diseases, Cardiovascular disease

- **Inclusion criteria**: This includes randomised controlled trials (RCTs) of phase II/III with either traditional group sequential design or adaptive designs in patients with CV disease. The trials were not included if the study stopped early because of other reasons, such as withdrawal of funding by funders during the study.
- **Population**: Patients with any conditions related to cardiovascular disease, which is defined according to ICD-10 Codes.
- **Study settings and locations**: Clinical trials conducted in any setting were included in this review, including hospital clinics, surgeries and primary care.
- **Interventions**: Any types of interventions used in trials were included, such as drugs, different dosages, medical devices and biomarkers.
- **Outcome measures**: Any CV related comorbidity, mortality and biomarkers were included as primary outcomes regarding to efficacy and safety endpoints. Composite outcomes were included as they are commonly used in CV trials. Quality of life measured by the questionnaires was also considered as an outcome.
- **Trial designs**: This includes traditional group sequential design and adaptive design.

Including trials with these designs posed a challenge as “adaptive” or “group sequential” was not commonly included within the title, abstract or key words of studies. To overcome this, we reviewed the reference list of retrieved articles to identify other eligible studies, such as the review paper of adaptive design [38] and the methodological systematic reviews of group sequential randomised controlled trials [39]. We included all studies with the words “interim analysis” in abstract in order to capture all possible trials. In addition, trials that had pre-specified interim analysis without a clearly reported stopping rule were carefully considered.

Two authors independently reviewed all titles and abstracts from the search results to identify articles that met the inclusion criteria. Selected studies was compared, and disagreement was resolved by discussion and consensus. If any of the eligibility criteria were not met, the article was excluded. There are common practice that the results of one trial are often published for more than one papers. We paid more attention to ensure that the main paper was selected. Articles finally selected for review were checked to avoid inclusion of data published in duplicate. The risk of bias summary for each included study and risk of bias graph were produced using Review Manager 5.4.

### Data synthesis

Relevant data were collected from baseline patient characteristics of clinical trials, trial demographics and trial design features, and was presented by the two types of clinical trial designs. Patient baseline characteristics included age and sex. Clinical trial demographic characteristics were descripted as year of study published, financial resources, country of chief investigator, the number of study centres/sites, primary outcome measure and trial population. In trial design characteristics, sample size, phase of trials, purpose of trial (safety, efficacy), frequency of interim analyses, mean/median follow-up, the number of arms, domain of intervention (drug, medical device), the reasons for early trial stopping (such as, safety, futility, efficacy or others), and type of statistical stopping rules were reported. For adaptive design, the type of adaptive was also reported for each study.

Descriptive statistics was used to provide an overview of how group sequential designs and adaptive designs were presented. Continues variables were summarised as mean (SD)/median (IQR) and categorical data were presented as count (percentage).

## Results

**Fig. 1:**
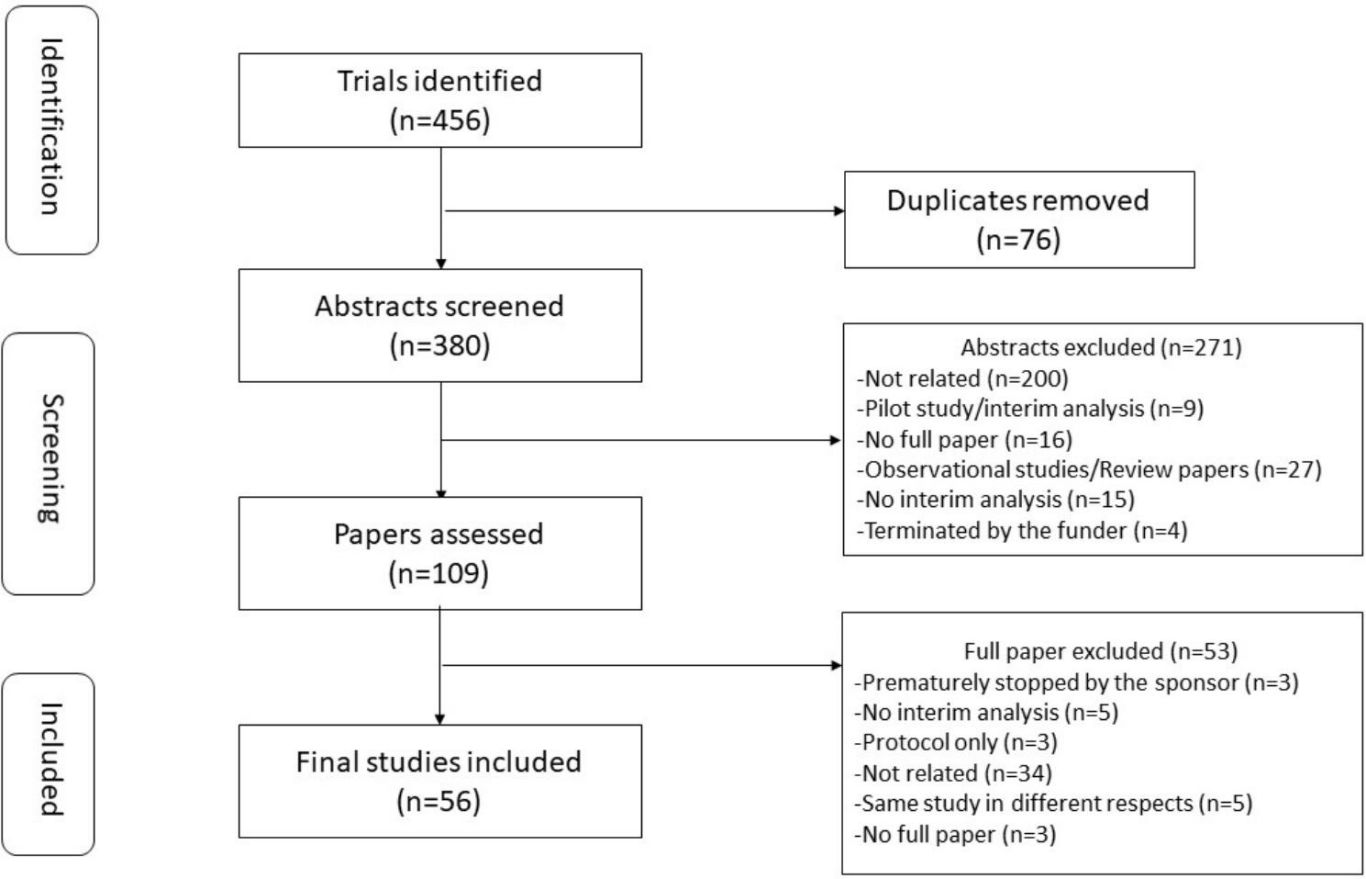
Flow diagram of the search strategy and studies identified from systematic review

The studies selection process and results are shown in Fig. 1. Of 456 articles found in the initial search, 109 were retrieved for more detailed evaluation and 56 studies were identified. The mean age was 66 years and 69% were male. The biggest trial contains 24,335 patients conduced in 4 countries with 625 centres and the smallest trial was a single centre, randomised controlled trial for 30 patients.

Most of the trials were multicentre (76% of the trials > = 50 sites), two-arm (74%) with a large sample size (67% of the trials > 1,000) and were led by the United States of America (28 (50%)) and Europe (16 (29%)). The number of trials with a median study period greater than or equal to 2 years were 27 (58.2%) and majority of the trials were funded by commercial companies 44 (78.6%). DSMB was almost included all trials (96%) (Tables 2 and 3).

**Table 2:** Summary of the clinical trial demographic characteristics

	Traditional group sequential design (n = 43)		Adaptive design (n = 13)	
	No.	Percentages	No.	Percentages
**Funders**				
Public	9	20.9%	2	15.4%
Commercial (company)	33	76.7%	11	84.6%
None	1	2.3%	0	0
**Country** (chief investigator)				
USA	18	41.9%	10	76.9%
European	13	30.2%	3	23.1%
Others*	12	27.9%	0	0
**Centres/sites**				
1	2	4.7%	1	7.7%
2–49	5	11.6%	3	23.1%
50–99	8	18.6%	4	30.8%
100–199	6	14%	2	15.4%
200–299	4	9.3%	2	15.4%
300–499	4	9.3%	0	0
500–799	7	16.3%	1	7.7%
800-1,100	7	16.3%	0	0

* Japan, Canada, Norway, Singapore, Thailand, Denmark, Sweden and Australia

**Table 3:** Summary of the clinical trial design characteristics

	Traditional group sequential design (n = 43)		Adaptive design (n = 13)	
	No.	Percentages	No.	Percentages
**Sample size**				
< 200	4	9.3%	2	15.4%
200–499	0	0	2	15.4%
500–999	5	11.6%	4	30.8%
1,000–4,900	14	32.6%	1	7.7%
5,000–9,999	9	20.9%	2	15.4%
> 10,000	11	25.6%	2	15.4%
**Trial type**				
Phase II	1	2.3%	4	30.8%
Phase III (or IIIb)	20	46.5%	8	61.5%
Not available	22	51.2%	1	7.7%
Study type*				
Efficacy	33	76.7%	7	53.8%
Safety	6	14%	0	0
Both	4	9.3%	6	46.2%
**Trial arms**				
2	37	86%	8	61.5%
3	4	9.3%	3	23.1%
>=4	2	4.7%	2	15.4%
**Intervention**				
Drug	34	79.1%	8	61.5%
Surgery	8	18.6%	1	7.7%
Biomarker	0	0	1	7.7%
Device	1	2.3%	3	23.1%
**Follow-up** (mean/median) (years)				
< 2	19	44.2%	10	76.9%
>=2	24	55.8%	3	23.1%

* The study type was determined based on the primary objective of trial (study purpose)

### Trials with traditional group sequential design

Forty-three (76.8%) randomised clinical trials applied traditional group sequential design. The average patient age was 65 years old and 69% were male. Most of the trials were multicentre (65% of the trials > 100 centres/sites), two-arm (37 (86%)) and were conducted after year 2000 (83.7%) from the USA and Europe (18 (42%) for the USA and 13 (30.2%) for Europe). About 56% of the trials had a mean/median follow-up greater than or equal 2 years and 47% of the trials had a sample size greater than 5,000 patients. The smallest trial had fewer than 200 patients and the largest had over 10,000 patients. In addition, 79.1% of the trials were related with drug interventions, 76.7% of the trials evaluated efficacy of interventions. Most trials were funded from commercial companies (33 (77%)) (Tables 2 and 3).

In addition, 39 (90.7%) had pre-planned interim analysis and the other four were planned by DSMB. Most trials (79%) had 2 or more interim analyses. 67% of the trials stopped early, in which 5 (11.6%) were due to futility, 14 (32.6%) due to efficacy, 9 (20.9%) because of safety concerns and one for other reasons. The number of interim analyses was determined based on either by numbers of events (47%) (Such as, all-cause mortality and a composite end point of CV death and nonfatal myocardial infarction), number of patients recruited (12%) or time periods.

The commonly used stopping rule to terminate trials was the O’Brien- Fleming-type alpha spending function (10 (23.3%)). 7% of the trials used the O’Brien-Fleming group sequential boundaries and 7(16.3%) used the Haybittle and Peto (or modified) type of stopping rule. Over 50% of the trials used other stopping rule methods or did not make it clear for which methods were used (Table 4). One trial made the decision to increase study time due to slower recruitment after the interim analyses, and in another trial, the trial stopped early because of publishing confidential interim data by the sponsor.

**Table 4:** Detailed features of traditional group sequential and adaptive designs

Traditional group sequential design		Numbers (n = 43)	Percentages (76.8%)
How was interim analyses planned	Pre-planned	39	90.7%
	Pre-planned by DSMB	4	9.3%
Frequency of interim analyses planned	N = 1	9	20.9%
	N = 2	16	37.2%
	N > 2	18	41.9%
How was the interim analysis set	Time	15	34.9%
	Patients	5	11.6%
	Events	20	46.5%
	NA	3	7%
Pre-planned decision rule	The O’Brien-Fleming group sequential boundaries	3	7%
	Haybittle and Peto type of stopping rule	7	16.3%
	An alpha spending function (Lan and DeMets): the O’Brien- Fleming-type	10	23.3%
	Triangular sequential design (A Wang-Tsiatis group sequential design)	2	4.7%
	Asymmetrical group sequential (a Peto-type boundary)	1	2.3%
	Others	9	20.9%
	NA	11	25.6%
Was the trial stopped early	Yes	29	67.4%
	No	14	32.6%
Reason for stopping early	Futility	5	11.6%
	Efficacy	14	32.6%
	Safety	9	20.9%
	Others	1	2.3%
**Adaptive designs**		Numbers (n = 13)	Percentages (23.2%)
How was interim analyses planned	Pre-planned	13	100%
Frequency of interim analyses planned	N = 1	9	69.2%
	N = 2	2	15.4%
	N > 2	2	15.4%
How was the interim analysis set	Time	0	0
	Patients	10	76.9%
	Events	2	15.4%
	NA	1	7.7%
Was the trial stopped early	Yes	7	53.8%
	No	6	46.2%
Reason for stopping early	Futility	4	30.8%
	Efficacy	2	15.4%
	Safety	-	-
	Others (slow enrollment)	1	7.7%
Type	Methods		
Re-estimation of sample size	Bayesian adaptive approach	2	15.7%
	Simulation	2	15.7%
	Others	2	15.7%
	NA	2	15.7%
Response-adaptive randomization	Bayesian approach	1	7.7%
Dose-Response	Bayesian method	1	7.7%
Enrichment	Bayesian method	1	7.7%
Two-stage adaptive	Simulation used to assess the type I error. A group sequential approach with efficacy boundary based on a gamma (− 10) α spending function.	1	7.7%
Seamless phase IIb/III	Simulation used to assess the type I error. A truncated Levin-Robbins sequential elimination procedure for selecting a right dose.	1	7.7%

NA: not available

Risk of bias assessment is provided in Supplementary Fig. 1 and Supplementary Fig. 2. Of the 43 trials, 20 had low risk of selection bias (random sequence generation) and most of the trials (92%) had low risk of selection bias of allocation concealment. Blinding (performance bias) domain had the highest rate of high-risk of bias (21%). Unclear risk of bias was high in attrition bias (67%) and other bias (70%).

### Trials with adaptive design

**Fig. 2:**
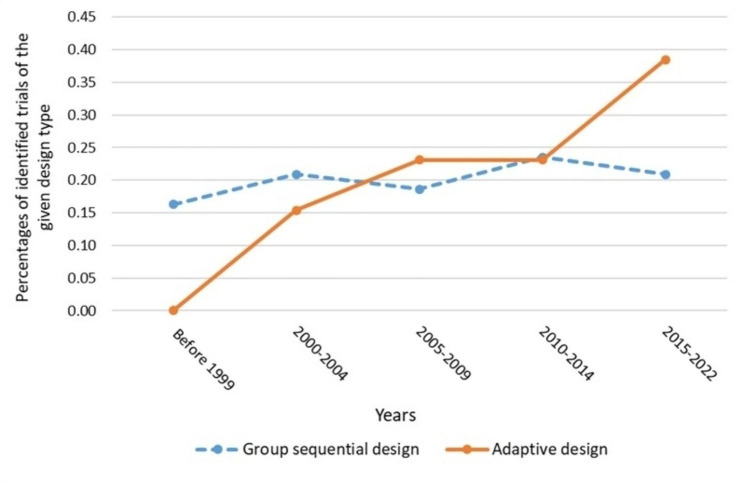
Percentages of identified trials between two groups

Thirteen (23.2%) randomised clinical trials applied the adaptive design. The average patient age was 66 years old and 68% were male. All trials were conducted after year 2000 in which 38.5% were after year 2015. Most of the trials were multicentre (69% of the trials > 50 centres/sites), two-arm (8 (61.5%)) and 23.1% of the trials had a mean/median follow-up greater than or equal to 2 years. All trial investigators were from either the USA (10 (76.9%)) or Europe (3 (23.1%)). 61.5% of the trials were related with drug interventions and 23.1% with medical devices. The number of trials tested for efficacy of interventions were 7 (53.8%) and most trials were funded from commercial companies (11 (84.6%)). The percentage of phase II trials was higher in adaptive trials (2.3% of the trails with the traditional sequential design vs. 30.8% in adaptive trails) (Tables 2 and 3). Adaptive designs have been increasingly used in trials in cardiology (from 0 (0%) before 1999 to 5 (38.6%) after 2015. The percentage was calculated by dividing the number of adaptive trials before 1999 (or after 2015) by the total number of adaptive trials identified in this review (Fig. 2).

All trials had at least one interim analysis because of nature of adaptive design. Of the 13 adaptive clinical trials, 9 (69%) had one interim analysis, 53.8% of the trials stopped early, in which 4 (30.8%) were due to futility, 2 (15.4%) for efficacy and one because of slow enrolment. Frequency of interim analyses was mainly determined by the number of patients (76.9%) and the number of events (15.4%). 54% of the trials stopped early, in which 4 (30.8%) were due to futility, 2 (15.4%) for efficacy and one because of slow enrolment. In addition, 8 (61.5%) used re-estimation of sampling size where 4 trials used simulation procedure including two Bayesian approach, and the other five used each of the following design: response-adaptive randomization with Bayesian method, dose response using Bayesian method, adapted enrichment, adaptive two-stage design, and seamless phase IIb/III (Table 4).

Risk of bias assessment is provided in Supplementary Fig. 3 and Supplementary Fig. 4. Of the 13 trials, 8 had low risk of selection bias (random sequence generation) and most of the trials (92%) had low risk of selection bias of allocation concealment. Blinding (performance bias) domain had the highest rate of high-risk of bias (38%). Unclear risk of bias was high in attrition bias and other bias (69% for both).

### Trial reporting

Majority of the trials (95%) with traditional group sequential and adaptive designs did not clearly indicate if study was following CONSORT guidelines [https://www.consort-statement.org/]. In most of studies the name of the design was not indicated in either abstract, title or key words of studies. Of the 12 adaptive trials excluding an ongoing trial, 3 (25%) reported “adaptive” in abstract or title, 7 (58%) described the design in method section, 5 (42%) indicated which software was used and 47% of the trials provided detailed information of implementation of the adaptive process on simulation including Bayesian adaptive approach used to define the stopping rules. Trials with traditional group sequential design, 16 (37.2%) reported “interim analysis” in abstract of studies.

## Discussion

In this review, we found that adaptive trials have been increasingly used in cardiovascular research. The reporting of these trial designs needs improving, which was in agreement with the findings of other study in oncology [40].

Trials with traditional group sequential design, frequency of pre-planned interim analyses plays an important role. One study recommend that the number between 4 and 8 interim analyses seems to be sensible in practice [9]. However, we observed that how frequency of interim analyses was determined was not clear at the pre-planned design stage. This issue has been discussed by McPherson, K. [41]. In addition, in most of the trials the number of interim analyses were determined based on the number of events, rather than numbers of patients, which might be due to the nature of study endpoints used in cardiovascular research field, such as mortality and combined CV events with mortality.

To stop trials early with traditional group sequential design, stopping rules are normally required. The study by Tyson, J.E. recognised that stopping guidelines are often vague [42].This finding was consistent with the current review, in which about 26% of the trials did not make it clear which statistical stopping rules were used. In this review, 67% of the trials stopped early. Trials did not stop early because no stopping boundary was crossed. Some studies can ran its full course as there were no significant differences seen early between the interventions. However, stopping trials early could cause bias in data analysis when the trials are still running [11, 43]. The benefits and challenges for stopping a trial early in cardiology were discussed previously [11].

This review found that various adaptive designs have been applied in trials in cardiology. Amongst adaptively designs trials, one of the most frequently used design is sample size re-estimation (62%). It is not uncommon in cardiovascular clinical trials to increase either recruitment rate or follow-up duration during the study because observed event rate was much less than anticipated [44, 45]. Sometimes sample size requires re-estimation after the interim analysis because of inaccurate estimated parameters used in the original sample size calculation [46]. In recently published review of adaptive clinical trials [29], 8% of trials were used sample size re-estimation. This difference is partly due to the fact that the percentage was calculated in a way that the number of group-sequential designs was included in the denominator. It might also be explained by the lack of cardiovascular trials included in the review.

Maintaining blinded interim analysis and employing DSMB are strongly recommended by both the EMA and the FDA. The reporting of blinded interim analysis was not clear in this review, this is similar to the study conducted from adaptive review [29]. However, we found that DSMB was almost included all cardiovascular clinical trials for reviewing data generated from interim analysis to assess if the trials should stop early and if any changes were required. In contrast, only 32% of the trials reported an independent data monitoring committees involved in the adaptive review.

Methodology used in adaptive trials has been studied partly by Mehta C [22]. Simulation including Bayesian approach is often used to facilitate the design and data analysis. The review found that the reporting how simulation was implemented to facilitate the trials was not clear. Only 53% of the trials provided detailed information. In addition, the reporting “adaptive” in either abstract or title was low. However, trial reporting with adaptive design looks encouraging. Extension statement of the adaptive designs CONSORT [47] has provided the updated guidance on reporting adaptive design. We noted that after the statement was issued one trial reported the study design accordingly using “adaptive” in the abstract and provided detailed information about the design and analysis.

Adaptive trials are increasing [29, 38]. The review found that all trials were conducted after year 2000, and 39% were after year 2015. A very recent study published in year 2021 showed that an adaptive trial design potentially increased success rates of the clinical trials by 4% points, and that it could save development costs for a new drug from 2.6 to 2.2bn USD [48]. Adaptive design, such as adaptive enrichment takes into account individual differences in trials [49]. The design could be beneficial for cardiovascular clinical trials as most patients are elderly where polypharmacy and multiple co-morbidities increase the complexity of trials. The design could lead to a more personalized therapeutic approach and better results in HFpEF clinical trials [50].

The review has several limitations. Despite the effort to search the related papers, we believe not all the papers are included in the review because of poor reporting of the designs in the studies, the key words, such as “adaptive” was not commonly found in neither title nor abstract of studies, as the results, it is inevitable that some papers have been missed. However, we believe that this study provides a broad review of how these designs were applied in cardiovascular randomized clinical trials, particular for trials with adaptive design. Furthermore, the participants in some studies were mixed of CV disease and other type of diseases. In addition, traditional group sequential design is defined as an adaptive design in some cases, which means that there is a chance that this design is grouped as an adaptive design by the authors. However, each trial with adaptive design was carefully checked to ensure classification as per criteria.

## Conclusion

We believe trials with adaptive design will continue to grow in cardiology. Reporting of adaptive designs in cardiovascular randomized clinical trials is inadequate and needs to improve.

### Electronic supplementary material

Below is the link to the electronic supplementary material.

Supplementary Material 1

Supplementary Material 2

## Data Availability

The datasets used and/or analysed during the current study available from the corresponding author on reasonable request.
