# Supplementary material for: A systematic review of randomised controlled trials with adaptive and traditional group sequential designs – applications in cardiovascular clinical trials
Source: BMC Med Res Methodol. 2023 Sep 7;23:200. doi: 10.1186/s12874-023-02024-1 (PMC10483862; doi:10.1186/s12874-023-02024-1)
Supplement: Supplementary file 1 — Supplementary Material 1 [file 12874_2023_2024_MOESM1_ESM.docx]

Online supplementary materials

**Manuscript title: A systematic review of randomised controlled trials with adaptive and traditional group sequential designs – applications in cardiovascular clinical trials**

Jufen Zhang (PhD), Christy Saju

Supplementary Figure 1. Risk of bias graph (trials with group sequential designs).

Supplementary Figure 2. Risk of bias summary for each included study (trials with group sequential designs).

Supplementary Figure 3. Risk of bias graph (trials with adaptive designs).

Supplementary Figure 4. Risk of bias summary for each included study (trials with adaptive designs).


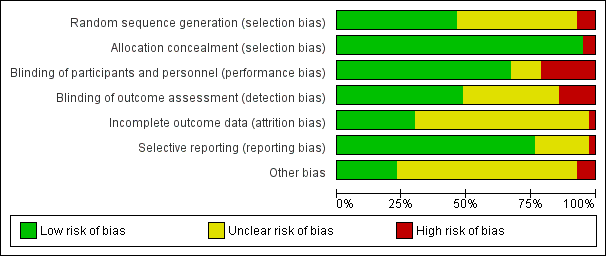


Supplementary Figure 1: Risk of bias graph (trials with group sequential designs)


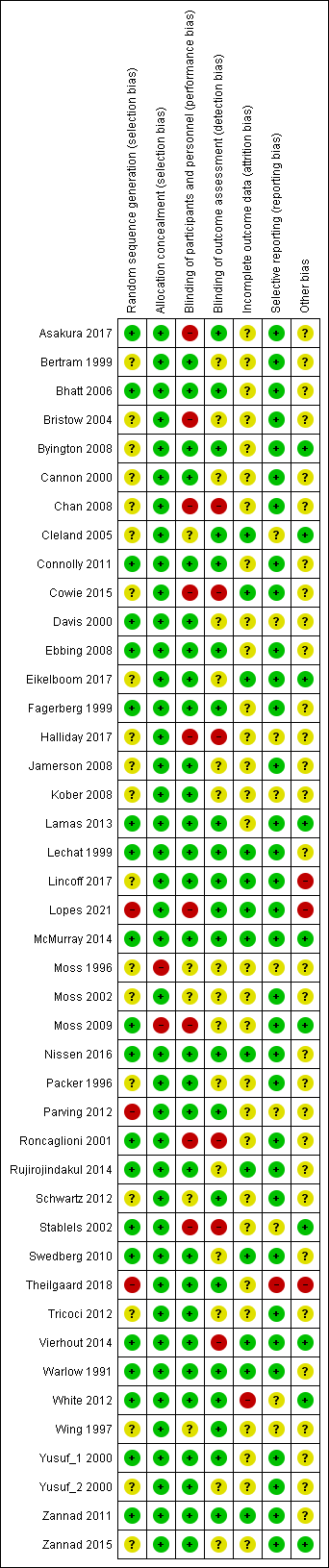


Supplementary Figure 2: Risk of bias summary for each included study (trials with group sequential designs)


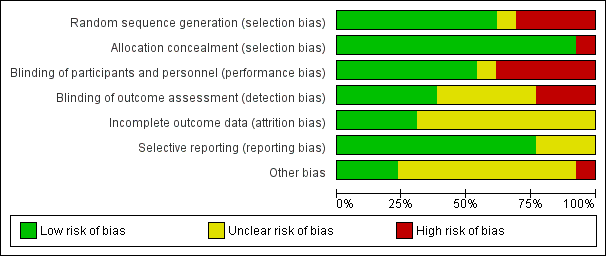


Supplementary Figure 3: Risk of bias graph (trials with adaptive designs)


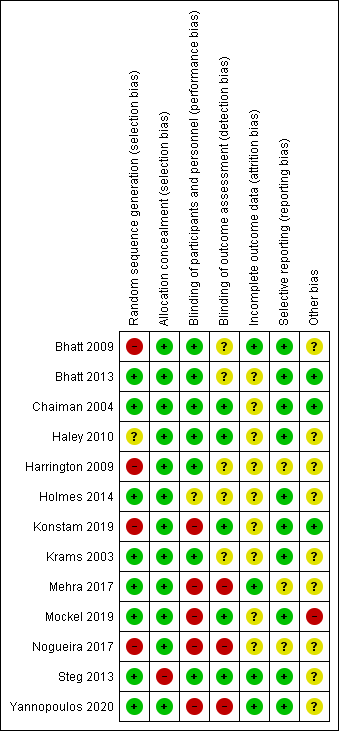


Supplementary Figure 4: Risk of bias summary for each included study (trials with adaptive designs)
